# From pixels to cell types: a comprehensive review of computational methods for spatial transcriptomics deconvolution

**DOI:** 10.1186/s44342-025-00055-2

**Published:** 2025-10-31

**Authors:** Jahanzeb Saqib, Junil Kim

**Affiliations:** 1 Department of Bioinformatics, Soongsil University, 369 Sangdo-Ro, Dongjak-Gu, Seoul, 06978 Republic of Korea; 2 School of Systems Biomedical Science, Soongsil University, 369 Sangdo-Ro, Dongjak-gu, Seoul, 06978 Republic of Korea

**Keywords:** Single cell RNA-seq, Spatial transcriptomics, Cell type deconvolution, Deconvolution algorithms, Probabilistic modeling, Deep learning, Bayesian inference, Non-negative matrix factorization (NMF), Graph-based modeling, Transformer based models

## Abstract

Spatial transcriptomics technologies have significantly enhanced the analysis of gene expression profiles by retaining the spatial information of intact tissue sections and enabling the possibility of a more profound comprehension of tissue structures and cellular relationships. Despite this, most platforms have limited resolution, and at numerous capture spots, multiple signals from various cells are present, requiring deconvolution, a set of computational steps to deduce the underlying cellular composition. Over the last few years, a range of algorithms has been proposed to address this problem, each employing distinct computational principles and processing paradigms. The present review seeks to present a comprehensive analysis of twenty such algorithms, focusing on their methodological foundations. We contrast the underlying computational algorithms, modeling methods, and data processing pipelines that underlie them, and how they deal with external references, noise and sparsity in the data. By drawing out the conceptual as well as technical foundations of each algorithm, we aim to provide researchers a complete and hands-on grasp of the computational landscape of spatial transcriptomics deconvolution. This review is a methodological handbook to enable deep understanding of current deconvolution methods to develop novel strategies and help in selecting or applying these existing tools for different biological contexts.

## Introduction

Spatial transcriptomics (ST) allows scientists to examine gene expression throughout tissues while maintaining spatial information about their original locations, thus enabling researchers to study intricate biological systems, including developmental progression and disease environments and tissue diversity [1]. The current ST technologies divide into imaging-based and NGS-based methods for molecular detection [2–4]. The two distinct technological classes differ through their resolution and transcriptome coverage levels and data throughput, which makes each platform suitable for different research applications.

Fluorescence microscopy enables imaging-based methods to detect mRNA molecules in tissue sections with precise spatial resolution. These methods provide single-cell or subcellular resolution, but their capacity to profile transcripts simultaneously remains limited. Single-molecule fluorescence in situ hybridization (smFISH) [5, 6] serves as the basic imaging-based method, using fluorescent probes to detect particular mRNAs. While effective for gene identification, traditional smFISH is insufficient for transcriptome-wide profiling. Sequential FISH introduced automated hybridization and imaging, allowing dozens of genes to be profiled with less manual labor, though expanding gene numbers still requires additional cycles. Combinatorial FISH with binary barcoding [7] addressed this limitation by assigning barcode sequences across imaging rounds, enabling detection of hundreds to thousands of genes in one experiment, but requiring advanced optical and decoding systems.

The NGS-based spatial transcriptomics approach extracts RNA molecules from permeabilized tissue sections by utilizing spatially barcoded oligonucleotides that bind to a permeabilized tissue surface [8–10]. The oligonucleotides bind to poly(A) tails of mRNAs so that sequencing libraries can be built that maintain gene information alongside spatial coordinates. The sequencing-based methods provide extensive transcriptome analysis while producing higher output rates, yet they trade off with reduced spatial precision. The spatial features known as spots or capture areas consist of mixed transcripts that originate from multiple cellular components. The capture spot dimensions in platforms like Spatial Transcriptomics and 10 × Genomics’ Visium [11] surpass cell size, so cells become indistinguishable from each other in the resulting data. The estimation of cell type composition in low-resolution spots requires computational deconvolution techniques, which need reference data along with specialized algorithms.

Multiple innovative techniques emerged to improve the spatial resolution of early NGS-based methods. Slide-seq [12] established the technique of placing barcoded microbeads on a coverslip to generate a dense spatial pattern, which enables near-single-cell resolution. Seq-Scope [13] improved mRNA capture resolution by using Illumina flow cell-based patterned oligonucleotide arrays, which provide submicron-scale measurement. Stereo-Seq [14] uses DNA nanoball (DNB) arrays to reach nanometer precision across extensive tissue areas while providing flexible viewing areas for high-resolution assessment. The technological advances in NGS-based spatial profiling have expanded its capabilities by merging improved resolution with complete transcriptome-wide unbiased capture capabilities.

Each platform presents trade-offs in resolution, gene coverage, scalability, and tissue compatibility. Despite this, the continuous development of ST technologies expands spatial biology’s capabilities for diverse biomedical fields. A shared challenge among NGS-based methods is low spatial resolution, which merges cellular signals into mixed capture units and reduces single-cell specificity.

Numerous computational methods have appeared in the scientific literature to solve this problem by deconvolving mixed signals while determining the cellular makeup of each spatial feature. The algorithms use external single-cell reference data together with spatial information and probabilistic models and machine learning approaches to identify the types of cells that exist in each spot. The deconvolution process transforms spatial data into meaningful biological insights, which allow researchers to map cellular niches and tissue microenvironments at high resolution.

The review presents an extensive analysis of computational approaches for spatial transcriptomics deconvolution with specific focus on methods designed for low-resolution data. The main goal is to deliver detailed practical information about active computational developments to researchers. The research functions as a methodological handbook that investigates the essential concepts and operational systems of modern deconvolution methods. The study presents the core principles and workflows of current deconvolution techniques. By examining their assumptions, strategies, and use across different biological systems, which help researchers to select the most suitable tools and inspire further development to address new challenges in spatial transcriptomics research.

## Main

**Fig. 1:**
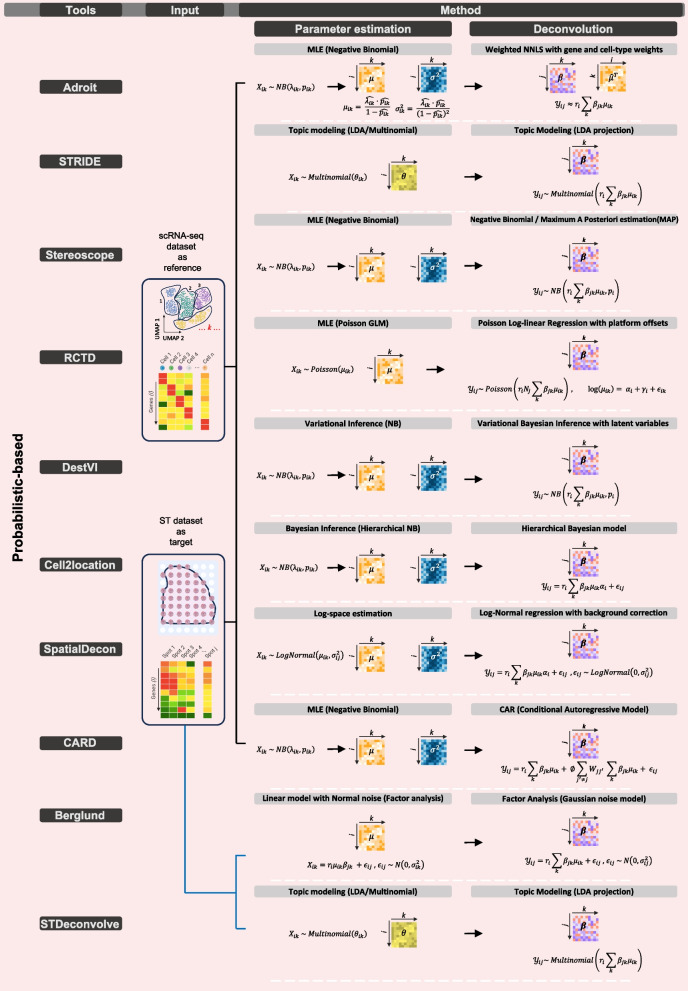
Mathematical formulations of probabilistic and regression-based deconvolution methods in spatial transcriptomics: The figure summarizes how probabilistic models (e.g., negative binomial, Poisson, Bayesian inference) and regression-based approaches (e.g., NNLS, Poisson GLM, factor analysis) utilize single-cell RNA-seq reference data to estimate cell type proportions in spatial transcriptomics. The equations illustrate key parameter estimation strategies applied during model training and deconvolution

**Fig. 2:**
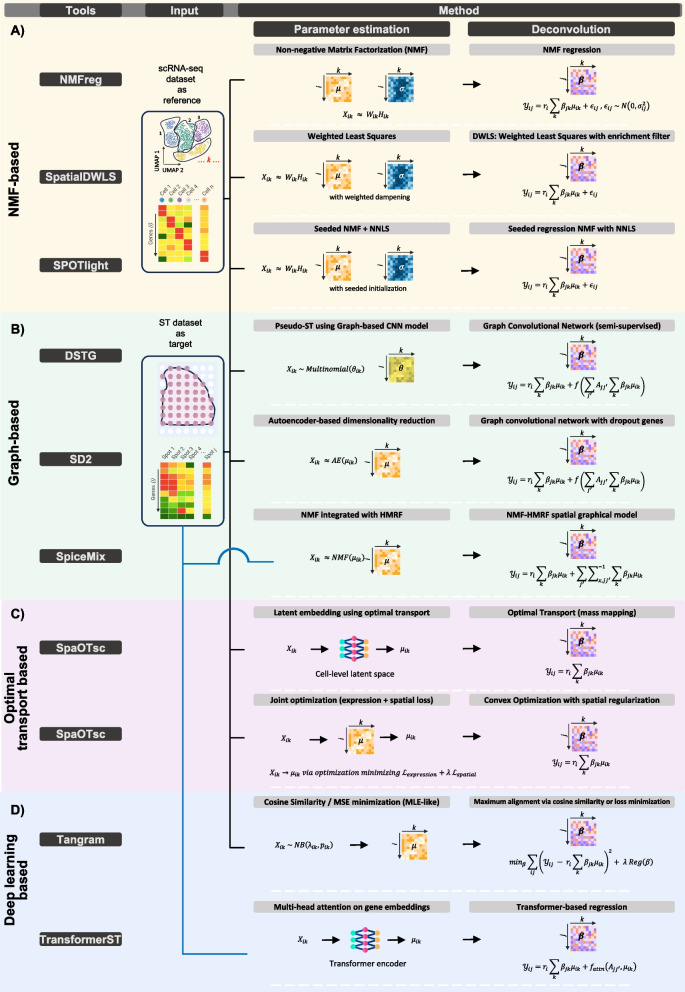
Mathematical frameworks of matrix factorization, graph-based, deep learning, and optimal transport-based deconvolution methods: This panel illustrates the core mathematical principles and workflows of four major classes of deconvolution methods. NMF (**A**), graph neural networks (**B**), optimal transport (**C**), and deep learning-based method (**D**). These models integrate spatial context through regularization, latent embeddings, or joint optimization of expression and spatial information to infer cell type distributions. UMAP plots represent the single-cell RNA-seq reference data, and the spatial transcriptomics slide represents the input as target data. Blue solid lines indicate reference-free methods using only spatial transcriptomics data, while black solid lines indicate methods that use both single-cell and spatial transcriptomics data as input

In recent years, various computational strategies have emerged to tackle the challenge of cell-type deconvolution in ST data. These approaches can be broadly classified into several categories, including probabilistic or stochastic models, non-negative matrix factorization (NMF)-based techniques, graph theory-driven methods, deep learning frameworks, and algorithms grounded in optimal transport theory. The visual guides in Figs. 1 and 2 provide a straightforward overview of mathematical concepts that validate these five categories. The first figure demonstrates how likelihood models (e.g., negative binomial, Poisson) and regression techniques (e.g., NNLS, Poisson GLM) enable researchers to calculate cell-type proportions from reference data. Figure 2 provides a detailed illustration of NMF and graph-based and optimal transport and deep learning methods by showing their core workflows and how they incorporate spatial context through regularization and embeddings and joint optimization of expression and spatial information.

The comparison between deconvolution algorithms in Table 1 includes their programming languages, modeling frameworks, essential characteristics, modality, platforms, Visium HD/tixel binning compatibility, and reference requirements. The table serves as a reference tool which helps to assess technical aspects and operational capabilities of particular tools. The comparison continues through Table 2, which presents method selection guidelines based on specific scenarios. The tool determines appropriate method classes for various analytical needs by using a mapping system which takes into account reference availability, spatial architecture strength, binned data management, computational resource constraints, and multimodal integration needs. By pairing the detailed comparison in Table 1 with the practical decision tree in Table 2, it aligns both conceptual understanding and application-specific needs.

**Table 1:** Overview and comparison of spatial transcriptomics deconvolution algorithms

No.	Name	Language	Model	Key features	Modality	Platform(s) validated/commonly used	Visium HD/Tixel binning support (8–16 µm)	Reference scRNA-seq required?
1	Adroit	R	Probabilistic	• Gene-wise technology bias correction • Smart gene weighting • Regularized linear modeling	NGS/spot-based	10 × Visium	No	Yes
2	STRIDE	Python	Probabilistic	• Topic modeling–based deconvolution • High-resolution spatial analysis • 3D tissue reconstruction capability	NGS/spot-based	ST, Slide-seqV2	No	Yes
3	SpatialDecon	R	Probabilistic	• Log-normal regression • Extensive cell profile libraries • Integration with GeoMx DSP	NGS/spot-based	GeoMx DSP, 10 × Visium	No	Yes
4	Cell2location	Python	Probabilistic	• High-resolution mapping via shared-location modeling • Integrated multi-dataset analysis with correction • Estimates relative and absolute abundances	NGS/spot-based	10 × Visium, Slide-seq	Yes (~ 8–16 µm)	Yes
5	DestVI	Python	Probabilistic	• Multi-resolution deconvolution • Joint modeling (scLVM and stLVM) • Automated downstream analysis pipeline	NGS/spot-based	10 × Visium, Slide-seqV2, Seq-Scope	Yes (~ 10 µm)	Yes
6	RCTD	R	Probabilistic	• Probabilistic cell mixture model • Platform effect normalization • Gene-level overdispersion handling	NGS/spot-based	10 × Visium, Slide-seqV2	No	Yes
7	CARD	R	Probabilistic	• Spatially aware deconvolution • High-resolution imputation • Reference-free capability	Hybrid (imaging + NGS)	seqFISH, ST, 10 × Visium, Slide-seqV2	No	Optional
8	Berglund	C + +	Probabilistic	• Reference-free deconvolution • Spatially aware factor modeling • Integrated multi-modal interpretation	NGS/spot-based	ST	No	No
9	STdeconvolve	R	Probabilistic	• Reference-free deconvolution • LDA-based cell type discovery • Data-driven feature and parameter selection	NGS/spot-based	ST, 10 × Visium, DBiT-seq, Slide-seq	Yes (10 ~ 25 µm)	No
10	Stereoscope	Python	Probabilistic	• Negative binomial modeling • MAP-based inference • No marker or pairing required	NGS/spot-based	ST, 10 × Visium, Slide-seq	No	Yes
11	NMFreg	R	NMF	• Integration via NMF • NNLS projection • Multiplet detection and confidence filtering	NGS/spot-based	Slide-seq	Yes (~ 10 µm)	Yes
12	SpatialDWLS	R	NMF	• Enrichment-based cell-type filtering (PAGE) • Dampened weighted least squares (DWLS) • Iterative deconvolution	Hybrid (imaging + NGS)	ST, 10 × Visium, seqFISH +	Yes (~ 51 µm)	Yes
13	SPOTlight	R	NMF	• Seeded NMF • scRNA-seq + spatial data integration (unit-variance normalization) • NNLS-based proportions	NGS / spot-based	ST, 10 × Visium	No	Yes
14	DSTG	Python	Graph	• Graph-based semi-supervised learning • Pseudo-ST data generation • Canonical correlation-based graph construction	NGS/spot-based	ST, 10 × Visium, Slide-seqV2	Yes (~ 10 µm)	Yes
15	SD^2^	Python	Graph	• Integration of dropout information • Spatially aware graph construction • Non-linear representation learning	Hybrid (imaging + NGS)	seqFISH + , MERFISH, ST, 10 × Visium	Yes (25 ~ 50 µm)	Yes
16	SpiceMix	Python	Graph	• Integrated spatial modeling (NMF-HMRF) • Spatially variable metagenes • Interpretable latent factors	Hybrid (imaging + NGS)	seqFISH + , STARmap, 10 × Visium	Yes (~ 30 µm)	No
17	SpaOTsc	Python	Transport	• Spatial reconstruction • Cell–cell communication • Gene–gene regulation • Spatial clustering • Multi-modal integration	Hybrid (imaging + NGS)	ISH, seqFISH + STARmap Slide-seq	Yes (~ 10 µm)	Yes
18	novoSpaRc	Python	Transport	• Atlas-free or guided reconstruction • Structure-aware mapping via GW-OT • Probabilistic and interpretable mapping	Hybrid (imaging + NGS)	FISH, MERFISH 10 × Visium Slide-seq	Yes (~ 10 µm)	Yes
19	Tangram	Python	Deep learning	• Single-cell spatial reconstruction • Multimodal integration • Anatomical context alignment	Hybrid (imaging + NGS)	smFISH, MERFISH, STARmap, smFISH, 10 × Visium	No	Yes
20	TransformerST	Python	Deep learning	• Reference-free single-cell resolution • Multimodal representation learning • Adaptive spatial graph modeling • Cross-scale super-resolution reconstruction	Hybrid (imaging + NGS)	MERFISH, ST, 10 × Visium, Stereo-seq, Xenium	Yes (~ 6–9 µm)	No

**Table 2:** Scenario-based guidelines for selecting deconvolution methods

Scenario	Recommended method class	Rationale
High-quality scRNA-seq reference available; moderate spot resolution (e.g., Visium)	Probabilistic (Cell2location, DestVI, RCTD)	High accuracy, uncertainty quantification
No suitable reference available; exploratory study in novel tissue	Reference-free (STdeconvolve, SpiceMix)	Flexibility, no dependency on external data
Strong tissue architecture with spatial gradients (e.g., brain layers, tumor niches)	Graph-based (DSTG, SD2)	Explicit use of neighborhood structure
Global tissue reconstruction from partial gene panels	OT-based (SpaOTsc, novoSpaRc)	Distribution alignment enables atlas-free reconstruction
Multi-omic integration (e.g., RNA + ATAC, histology) or fine structural resolution	Deep learning (Tangram, TransformerST)	Scalability, multimodal flexibility
Resource-constrained environment, large datasets, need for speed	Regression/NMF (SpatialDWLS, SPOTlight, NMFreg)	Computational efficiency and ease of use

The first group probabilistic methods includes tools like Adroit [15], STRIDE [16], SpatialDecon [17], cell2location [18], DestVI [19], RCTD [20], CARD [21], Berglund [22], STdeconvolve [23], and stereoscope [24]. These methods typically model the underlying data distributions and rely on likelihood-based inference mechanisms. The second category involves NMF-driven approaches, such as NMFreg [12], SpatialDWLS [25], and SPOTlight [26], where matrix factorization is central to the computation. Graph-based strategies, making up the third category, include tools like DSTG [27], SD2 [28], and SpiceMix [29], which utilize graph structures to capture spatial and cellular relationships. The fourth group utilizes optimal transport theory, with SpaOTsc [30] and novoSpaRc [31] being prime examples. Lastly, deep learning-based tools such as Tangram use neural networks and customized loss functions to infer cell-type proportions. Within this deep learning realm, transformer-based models like TransformerST [32] have also emerged, applying attention mechanisms and transformer architectures to spatial transcriptomics deconvolution. This review systematically explores these methodologies in detail in the following sections.

### Probabilistic model-based methods

#### AdRoit

AdRoit was originally developed for deconvoluting bulk RNA-Seq data consisting of multiple cell types. Since the bulk RNA-Seq platform may have varying effects on various genes, regression-based adaptive learning is employed to estimate gene-wise corrections. The algorithm models the gene-wise technology bias, cross-sample variability of genes, and cell type specificity. This scheme ensures that the accuracy of the deconvolution process can be improved by applying corrections to the individual characteristics of each gene.

The first step of AdRoit involves maximum likelihood estimation (MLE) to determine the mean and dispersion of each gene by considering the natural variability and distribution of gene expression levels across each cell type. The algorithm selects genes that provide essential information about cell type identity while disregarding genes with dominant large cell clusters. The algorithm standardizes cell type distribution in the single-cell UMI count matrix and determines the variance of each gene across all cells. The variance-stabilizing transformation (VST) enables AdRoit to perform data normalization and noise reduction while maintaining low computational complexity.

The second step in AdRoit involves the use of regularization to reduce the collinearity between cell subtypes that are very similar to each other. This is a common problem in complex tissues. The regularization term reduces the effect of statistical collinearity and makes it easier to distinguish between similar cell clusters while keeping enough sensitivity to separate them. This scheme achieves accurate and robust deconvolution for inferring complex cell compositions. In the last step, AdRoit employs a non-negative least squares regression model to deduce the cell compositions. For training purposes, it uses a weighted and regularized loss function. Moreover, a penalty term is added to regulate the complexity-accuracy trade-off.

#### Stride

STRIDE (spatial transcriptomics deconvolution by topic modeling). The method leverages topic modeling by utilizing Latent Dirichlet allocation (LDA) [33] a generative probabilistic model, to discover latent semantic structures, referred to as topics, from single-cell RNA sequencing data. These topics represent patterns of gene expression that are associated with different cell types to decompose cell types from spatial mixtures by training topic profiles from scRNA-seq data.

There are several key steps for the implementation of STRIDE. Initially, it identifies cell type-associated topics from annotated single-cell transcriptomes using LDA. To infer the cell type compositions for each location of spatial transcriptomics, the algorithm applies the pre-trained topic model. This is accomplished by estimating the topic-by-cell distribution and the gene-by-topic distribution from scRNA-seq data. An online variational Bayes (VB) algorithm [34] is used to summarize the topic-by-cell distribution to the cell-type-by-topic distribution. It executes several models with different topic numbers and evaluates the models by the accuracy of single-cell re-annotation to ensure the accuracy of the deconvolution process. Considering the accuracy of cell-type assignments for single cells, this algorithm selects the optimal topic number.

Subsequently, the pre-trained topic model is employed to deduce the topic distributions of each location in spatial transcriptomics. The cell-type fractions of each spatial location can be inferred by combining the cell-type-by-topic distribution and topic-by-location distribution. Furthermore, STRIDE can identify cell-type-related topics and underlying functions. This information may be exploited to integrate successive sections and reconstruct the three-dimensional architecture of tissues.

#### Stereoscope

The stereoscope enables the prediction of cell types through gene expression profiles instead of using specific marker genes. The algorithm integrates cell data from single cells with spatial data to generate tissue section maps of cell types. The algorithm uses negative binomial distribution to model both spatial and single-cell data because this distribution is typical for gene expression count data. The algorithm uses this assumption to deconvolve spatial data before identifying the cell type populations that produced the observed gene expression values at each spatial location.

The algorithm starts by characterizing cell-type expression profiles through single-cell data to enable deconvolution. The model-based method operates directly with raw count data because it eliminates the need for normalization procedures or other data transformations. The algorithm starts by applying a statistical model such as negative binomial distribution to single-cell RNA-seq data to calculate cell type-specific parameters. The algorithm determines maximum likelihood estimators (MLE) for these parameters through analysis of the provided single-cell data. The algorithm uses maximum a posteriori (MAP) estimation to deconvolve spatial data after it estimates parameters, including cell type gene expression rates and transcript observation probabilities from single-cell data. Stereoscope uses learned parameters together with MAP estimation to determine the cell type proportions at each spatial data capture location.

#### RCTD

RCTD (reference-based cell type decomposition) is a computational method originally developed for deconvoluting a high-resolution spatial transcriptomics Slide-seq. It is an extension of the Latent Dirichlet Allocation (LDA) probabilistic model, which is used to uncover underlying themes within large collections of text or documents. In the context of single-cell RNA sequencing, RCTD adapts this approach to identify and quantify cell types within mixed populations of cells based on their unique gene expression profiles.

RCTD assumes that individual reads are sampled from a randomly chosen gene from a randomly chosen cell type. It extends the LDA model by introducing platform effects and random effects that account for gene-specific overdispersion. The algorithm defines the predicted gene probability for each gene and cell type, using a Poisson model to approximate the distribution of gene expression levels.

RCTD estimates platform effects and uses Sequential Quadratic Programming (SQP) from DWLS package for MLE to optimize the log-likelihood of the model. The convergence of the algorithm is defined in terms of the L1 norm of the difference in weight vectors between iterations, with a default convergence threshold set to a small value to ensure precision. Not only this, but it also offers flexibility in model selection, allowing users to choose a value for the hyperparameter that controls the sparsity of the solution. The algorithm can operate in different modes, such as doublet mode and multimode, with the confidence in each cell type prediction determined by the fit of the model.

#### DestVI

DestVI is categorized as a multi-resolution deconvolution algorithm based on a Bayesian model. The focus of this method is to identify the continuum of cell types within spatial transcriptomics data. It captures the continuous variation of the transcriptome within cells of the same type. This method combines continuous sub-cell-type latent variations and discrete cell-type-specific profiles. It does this by using a conditional deep generative model.

The DestVI has two distinct latent variable models (LVMs), the single-cell LVM (scLVM) and the spatial LVM (stLVM). Both LVMs assume that the number of observed transcripts for each gene follows a negative binomial distribution, which is parameterized to account for the variability in gene expression. Comparatively, the scLVM captures discrete cell-type-specific profiles, whereas the stLVM captures continuous sub-cell-type latent variations and maps these onto the spatial data. The algorithm uses a joint representation of the single-cell and spatial data to produce high-resolution spatial characterization of the cellular structure of tissues.

DestVI operates within a Bayesian framework, which allows for the incorporation of prior knowledge and the estimation of posterior distributions of the latent variables. A conditional deep generative model is used to learn the latent variables that capture the continuous variation within cell types. The algorithm deconvolves the spatial transcriptomics data by estimating the proportions of each cell type in every spot and the cell-type-specific transcriptional state. Additionally, it includes a post-hoc analysis pipeline that facilitates guiding downstream analysis by highlighting the main axes of spatial variation. Cell-type-specific differential expression allows for the extraction of molecular signatures that characterize a given tissue segment or distinct locations within the same tissue.

#### Cell2location

Cell2location is based on a Bayesian model designed to map cells on tissue. It estimates the relative and absolute abundances of cells by decomposing gene expression counts measured across spatial locations into a set of predefined reference cell types. This technique is derived by representing it as a graphical model, and it requires input data that includes gene expression counts obtained from several spatial regions. Considering the technical effects of batching, the algorithm jointly models the data across multiple spatial datasets. For 10X Visium data, the gene expression count matrix can be directly obtained from the 10X SpaceRanger program and applied as an input to Cell2location. It is essential to generate count matrices for other technologies and then transform them into the Scanpy data format.

The cell2location model estimates the absolute abundance of predefined cell types at each spatial location by modeling gene expression counts using a negative binomial distribution, which effectively captures the over-dispersion often present in spatial transcriptomic data. The expected expression at each location is computed as a function of several components: gene-specific technology sensitivity, additive background noise, and a location-specific scaling factor for RNA detection efficiency. A critical strength of the model lies in its factorized hierarchical prior on the cell abundance variable, which captures structured co-localization patterns across spatial locations by introducing latent groups of cell types. This design allows the model to borrow statistical strength across regions, improves sensitivity to detect rare or fine-grained cell types, and regulates the expected sparsity of cell types per location, which is crucial for tissues with variable cellular composition. The hyperparameters governing these priors, especially the expected number of cells per location and expected number of cell types per location can be estimated directly from histological images or derived from image-based cell segmentation pipelines. These values are then used as inputs to define Gamma-distributed priors on cell abundance. Users are also encouraged to perform prior predictive checks to validate the adequacy of their hyperparameter settings.

In addition to its hierarchical prior structure, cell2location is equipped to jointly model multiple spatial transcriptomic batches or experiments, allowing for direct comparison of cell abundance across slides while accounting for batch-specific variation in RNA detection sensitivity. To support this, the model introduces a regularization hyperparameter that controls the strength of within-experiment normalization. The model employs approximate variational inference implemented through GPU-accelerated PyMC and Pyro within the scvi-tools framework, which enables scalable and efficient training even on large datasets. The use of biologically informed priors on gene-specific effects, detection efficiency, and background contamination helps improve mapping accuracy. Overall, cell2location represents a robust and flexible framework for spatial deconvolution, especially suited for resolving rare, transcriptionally similar cell types in complex tissue architectures.

#### SpatialDecon

The SpatialDecon models background and utilizes log-normal regression to achieve better results than traditional least-squares methods. The algorithm generates paired data of highly multiplexed gene expressions, which finds its best application in cancer research. The algorithm identifies genes which cancer cells express at minimal levels to serve as targets for immune deconvolution in tumors. This technique serves as a mapping instrument for cell types within spatial gene expression data. The system enables the creation of cell profile matrices for different tissue types. The technique enables detailed cell population analysis of tissue samples and represents a major advancement in the field.

The algorithm employs log-normal regression to handle the natural unevenness and variability found in gene expression data. The algorithm works by modeling gene expression based on known cell-type profiles and simultaneously removing technical background noise. The algorithm includes an outlier detection process to improve estimation precision by removing gene-region combinations and provides statistical results with abundance estimate confidence intervals and p-values. The absolute cell counts emerge from SpatialDecon when nuclei counts become available, such as through GeoMx technology. The reverse deconvolution framework of the system separates gene regulation from compositional effects to detect genes whose variability exceeds cell-type mixing effects. The method enables precise quantitative analysis of intricate tissue microenvironments throughout space.

The method allows users to create cell profiles for unmodelled cell types. This method proves especially valuable in cancer research because cancer cells’ expression profiles are often unknown to scientists. The algorithm enables researchers to select and analyze cancer cell-only areas which leads to more precise tumor analysis.

#### CARD

The CARD algorithm integrates single-cell RNA sequencing data with spatial expression data through Conditional Autoregressive-based Deconvolution. The NMF model links the three matrices (single-cell expression profile, spatial expression profile, and cell-type composition matrix) to the CAR model which accounts for spatial correlation. The approach enables efficient borrowing of cell-type composition data between spatial locations to achieve better deconvolution results. A maximum likelihood framework with constrained optimization enables CARD to determine the cell-type composition matrix by maintaining non-negative elements during the estimation process.

The first step of CARD uses spatial transcriptomics data along with a single-cell RNA-seq reference to identify genes that help distinguish between cell types. The algorithm produces a reference matrix that contains typical gene expression values for each cell type. The model represents spatial data as a blend of reference profiles, which allows it to predict cell type proportions in each spatial area. The CAR model (The Gaussian case) is applied to enhance accuracy through spatial smoothing of estimates.

The CARD method stands out by using spatial correlation models to predict cell-type compositions and gene expression values in unmeasured tissue locations, which produces more accurate deconvolution results even with imperfect scRNA-seq references. The ability to predict spatial patterns allows researchers to generate detailed spatial maps with high resolution, which helps reveal tissue architectural details and discover genes that exhibit spatial expression. The CARD algorithm processes extensive datasets that combine thousands of genes with multiple spatial positions. The non-negative cell-type composition matrix receives fast multiplicative updating rules in each optimization iteration to achieve this efficiency.

The extended version of the CARD algorithm known as CARDfree, functions as a reference-free method for cell-type deconvolution. The CARDfree method requires scRNA-seq reference data for its operation, but it only needs a list of gene markers that identify cell types. CARDfree provides less precise results than CARD but detects overall tissue domain separation patterns when reference-based deconvolution becomes impossible in specific cases.

#### Berglund

The Berglund algorithm is a reference-free unsupervised factor analysis algorithm, which processes spatial transcriptomics data without requiring single-cell reference profiles. The algorithm analyzes approximately 6,750 tissue regions from prostate cancer sections to reveal distinct gene expression profiles that exist across normal and prostatic intraepithelial neoplasia (PIN) glands together with immune cells and stroma and cancer regions. The method’s primary advantage lies in its power to detect spatial patterns and genetic variations throughout regions where no distinct histopathological features exist. The algorithm employs a probabilistic approach to extract latent biological signals from data while performing spatial smoothing and supporting multiple sample analyses for complex tissue organization and tumor microenvironment research.

In the core implementation of the Berglund algorithm gene expression counts undergo Poisson factorization decomposition into multiple factors, which generate observed spot counts. The algorithm assigns each factor to contain both a gene expression profile together with spatial activity maps. During inference the algorithm runs a Monte Carlo Markov Chain (MCMC) process to determine latent variables through Gibbs sampling for conjugate priors and Metropolis–Hastings steps for non-conjugate parameters. The spatial activity of each factor exists independently for every spot and spatial coherence can be added through kernel-weighted field smoothing. The model includes optional features to include both experiment-specific expression scaling and spot-level normalization for depth and quality adjustments between samples.

The algorithm uses t-distributed stochastic neighbor embedding (t-SNE) for dimensionality reduction to make spatial gene expression patterns more interpretable in a low-dimensional color space. The spots undergo hierarchical clustering to determine spatial positions through Euclidean distances, which reveals distinct transcriptional domains within tissues. The color-coding system enables straightforward visualization of each cluster and its spatial mapping. The method performs biological interpretation by running pathway enrichment analysis to detect gene-level expression outliers through annotations from curated pathway databases. The method generates a similarity tree from factor expression profiles to determine tissue component relationships and uses read depth segments to compare gene expression with copy number variations against the reference genome. This comprehensive framework allows researchers to dissect spatial tissue complexity with high granularity and biological resolution.

#### STdeconvolve

The deconvolution of cell types in ST data sets does not require any reference for this algorithm. LDA, a generative statistical model from natural language processing (NLP), is used for this purpose. The gene counts matrix from the ST dataset is used as an input for the algorithm. The algorithm then performs a feature selection step to find the appropriate genes for deconvolution. These genes are expressed across a moderate number of pixels and are also diverse among the pixels (remove genes expressed in < 5% or in 100% of pixels). Genes that have either low or high pixel-wise expression are eliminated (retain significantly overdispersed genes; default: top 1000 to capture cell-type variation).

STdeconvolve algorithm calculates the optimal number of cell types to be deconvolved. Using LDA modeling, STdeconvolve assigns a probability distribution over multiple cell types to each pixel, which allows it to estimate the proportional representation of each cell type within the pixels. The algorithm generates two output matrices, which display deconvolved transcriptional profiles of cell types and estimated cell type percentages in each pixel. The matrices reveal how different cell types are distributed throughout the ST dataset while showing their transcriptional characteristics.

STdeconvolve has several advantages over other deconvolution methods that require references. It is independent of an appropriate dataset, making it particularly useful when such references are unavailable. Moreover, it can retrieve gene expression profiles that are particular to perturbations, enabling the identification of transcriptional changes caused by perturbations. The algorithm is adaptable and can be utilized with various ST dataset resolutions and technologies. The architecture of this system is specifically tailored to address the distinctive characteristics of ST data. These include the presence of a small number of cells and cell types in each pixel, the minimal influence of batch effects, and the probable variation in the distribution of cell types across pixels in tissues.

The probabilistic model–based methods use negative-binomial and Poisson–NB distributions to model spot counts because these distributions allow the generation of spot-level expression values from reference-learned parameters and cell-type mixtures followed by likelihood-based proportion estimation (AdRoit, Stereoscope, RCTD, cell2location, DestVI, STdeconvolve; see Fig. 1, middle panel, Parameter estimation). The two methods handle platform bias and over-dispersion and within-cell-type heterogeneity differently in their implementation. The system learns gene-specific corrections through AdRoit and it combines cell-type weights with regularization techniques to improve performance across different tissue types and between different platforms. The Cell2location model uses hierarchical priors for gene, location and technology-specific effects to perform variational inference and borrow strength between spots, which enables the detection of rare types and reduces noise in maps. The probabilistic framework of DestVI includes both continuous state variations within cell types and proportions of cell types, which results in increased computational requirements for modeling flexibility. As summarized schematically in Fig. 1 (and contrasted with the regression formulations shown on the right panel), the probabilistic formulations emphasize generative count modeling and posterior/likelihood-based estimation, whereas regression methods solve constrained linear mixing problems. The probabilistic models demonstrate excellent performance in simulated and real-world data analysis through principled uncertainty management, but (i) they need a suitable scRNA-seq reference and proper hyperparameter/prior selection and (ii) greater GPU/CPU and memory resources and wall-time than basic regression models, and (iii) they may produce biased estimates when the reference is mismatched or when gene panels are shallow, as reported in recent reviews and benchmarks [37–39].

### NMF-based

#### NMFreg

The NMFreg algorithm, also known as non-negative matrix factorization regression, maps scRNA-seq cell types onto Slide-seq data, the method designed to evaluate the contribution of each cell type to the RNA on a particular bead. There are two primary phases of the algorithm.

In the first phase, the previously annotated single-cell atlas data with cell type identities is employed to derive a basis in reduced gene space via non-negative matrix factorization (NMF). NMF is a set of techniques that decompose a matrix into two matrices, with the property that all three matrices contain only non-negative elements. This non-negativity makes the resulting matrices easier to inspect and leads to a parts-based representation because they allow only additive combinations.

When working with NMFreg, selecting highly variable genes is the first step for performing NMF on the single-cell data. Then, NMF is carried out using a predetermined number of factors. Subsequently, each factor is allocated to the cell type whose cells had the highest frequency of having the greatest loading on that factor. In the second phase of NMFreg algorithm, the non-negative least squares (NNLS) regression is used to compute the loadings for each bead in that basis. The loadings are then employed to determine the contribution of each cell type to the RNA on that bead.

To account for the presence of multiple cell types within a single spatial bead, NMFreg allows for the identification of mixed contributions by evaluating the L2 norm of each factor; any cell type whose loading exceeds 50% of the total norm is considered present. To ensure robustness, the algorithm is executed across a range of factor numbers and random initializations, and consistency is measured by the frequency with which a bead is assigned to the same cell type across runs. The final output is a high-resolution spatial map, in which each bead is annotated with one or more confidently inferred cell types, facilitating tissue-wide visualization of cellular organization.

The study considered an adult mouse single-cell cerebellum and an adult hippocampus scRNA-seq dataset to implement NMFreg. The method was configured with a threshold of 30 and a cutoff of 5 variable genes for the inclusion of bead in the analysis. Beads were assigned to cell types based on the identification of the first-level published clusters. There is a confidence thresholding stage in the algorithm as well. The algorithm was performed by employing only beads with at least 100 total transcripts, and NMFreg returned generic bead factor loadings. The result is a 72.6% reduction in the total number of beads called.

#### SpatialDWLS

SpatialDWLS is an approach built upon the dampened weighted least squares (DWLS), which was previously proposed for deconvolution of RNAseq data. The DWLS algorithm employs a weighted least squares technique to estimate the composition of cell types, where the weight is selected to minimize the overall relative error rate.

SpatialDWLS has two primary phases when applied to spatial transcriptomics. In the first phase, it identifies cell types that are likely to be present at each location. This is accomplished by performing an enrichment analysis using the parametric analysis of gene set enrichment (PAGE) technique. The marker genes can be discovered through differential expression gene analysis. Subsequently, the enrichment matrix is employed, using a threshold value of ES (enrichment score = 2), to select cell types that are likely to be present at each point.

In the second phase, the task is to estimate the cell type composition at each location. This is achieved by employing DWLS (differential weighted least squares) to estimate the fraction of each selected cell type. A cross-validation technique is used to determine the value of damping constantly to enhance numerical stability. To keep technical variance to a minimum, the same sets of weights and damping constant are used across spots within the same clusters.

After removing cell types that are predicted to be present at a low frequency, another round of deconvolution is conducted because the number of cells present at each spot is generally small. This is achieved by imposing an additional thresholding (min frequency = 0.02 by default).

Using a publicly accessible single-cell RNA-seq dataset, cell-type-specific gene signatures are obtained by applying SpatialDWLS. The simulated dataset is deconvolved using SpatialDWLS, which is based on these derived cell-type gene signatures.

#### SPOTlight

The SPOTlight algorithm performs unit-variance normalization to combine spatial transcriptomics (ST) data with single-cell RNA sequencing (scRNA-seq) data. The algorithm depends on seeded non-negative matrix factorization (NMF) regression to improve sensitivity and robustness through the use of cell-type marker genes.

The algorithm begins by using cell-type marker genes to establish NMF regression. The marker genes are obtained through the FindAllMarkers function in Seurat [35]. The initialization of topics in SPOTlight begins with the assignment of membership values between 1 and 0 for each cell in the topic. The algorithm achieves better accuracy through this initialization process.

The ST capture spots undergo deconvolution analysis through non-negative least squares (NNLS) in SPOTlight. The algorithm populates the coefficient matrix of capture locations while determining spot composition through cell type-specific topic profiles. The profiles show how genes distribute to define states or cell types. The algorithm accepts both paired and unmatched ST and scRNA-seq raw count matrices as input through its unit-variance normalization process.

The algorithm achieves better results through different input parameters that include scRNA-seq protocols and sequencing depth and cell numbers. The accuracy remains unaffected by lowering the sequencing depth. The training of SPOTlight model becomes more efficient through the use of cell numbers per cell type to reach an optimal balance between computational time and performance.

The NMF family operates with a basic interpretable method that combines each spot count with reference cell-type profiles through non-negative linear mixing followed by NMF + NNLS or weighted least-squares optimization after marker-based enrichment for fast and scalable results at thousands of locations. The main distinction between methods exists in their approach (see Fig. 2A) to maintaining proper fit and preventing false positives. NMFreg is the archetype, mapping Slide-seq beads with an NMF basis learned from scRNA-seq but with limited validation beyond Slide-seq, underscoring potential platform sensitivity. The SpatialDWLS method begins with PAGE enrichment screening to eliminate improbable cell types before it runs dampened weighted least squares analysis with a cross-validated damping constant that functions for all spots in a cluster to achieve better practical results. The SPOTlight algorithm performs seeded NMF to produce sparse cell-type-specific topics, which then undergoes NNLS deconvolution that maintains stability when using small single-cell references and identifies expected cortical and hippocampal structures. Collectively, these approaches are attractive when one wants transparent linear mixing, speed, and easy integration of marker lists or scRNA-seq references at the same time, they inherit the usual caveats performance hinges on reference quality, linear mixing and non-negativity assumptions, and (relative to Bayesian/probabilistic tools) they offer limited native uncertainty quantification or modeling of platform effects.

### Graph-based

#### DSTG

The DSTG (deconvoluting spatial transcriptomics data through graph-based convolutional networks) algorithm performs spatial cell type identification and high-level segmentation through graph-based convolutional networks (GCNs). The semi-supervised GCN model uses scRNA-seq data to precisely determine the composition of spatial transcriptomics (ST) data. The method specifically addresses the discrete and heteroskedastic nature of ST data.

In the beginning, synthetic pseudo-ST data is constructed by employing scRNA-seq data, which acts as the learning basis for DSTG algorithm. Based on the nearest neighbors shared by both the pseudo-ST and real-ST data sets, it subsequently learns a link graph of spot mapping. Using pseudo and real-ST data, the link graph captures the association between gene expression patterns and cell compositions across spots in both data types.

With two inputs, the data matrix of combined pseudo-ST and real-ST data and the spot similarity graph structure learned from the link graph, DSTG predicts the cell type compositions of the real-ST data. By employing these inputs, the algorithm constructs multiple convolutional layers, resulting in efficient training. The real-ST data will be left unlabeled and used for prediction, while the pseudo-ST data is randomly divided into test, validation, and training sets.

The training process of DSTG includes canonical correlation analysis to reduce input data dimensions. The algorithm identifies closest neighbors in real-ST data before creating a final link graph through an adjacent matrix. The link graph contains spot relationships that derive from their nearest neighbor positions.

The cellular composition learning from ST data becomes possible through DSTG because it combines variable genes with graphical structures by using nonlinear propagation in each layer. The system works with multiple normalization approaches while maintaining its ability to process ST data with changing genes and varying spot numbers.

#### SD2

The spatially resolved transcriptomics deconvolution (SD2) aims to characterize gene expression profiles while keeping spatial information about the tissues’ contexts. The SD2 technique integrates spatial information of ST data and effectively addresses the issue of dropout, which is typically referred to as a hurdle in single-cell RNA (scRNA-seq) analysis.

The SD2 method initiates by generating a pseudo-ST spot at random, with an expression vector that represents the proportion of a particular cell type. The resolution of the ST data is associated with the selection of these spots. After the training phase, the algorithm takes each spot’s dimensions as an attribute and uses them to capture the embedded space. It creates an unweighted graph with all real and pseudo-ST spots as nodes. The transcriptional and spatial levels define the graph edges, which identify the mutual nearest neighbors between the spots.

SD2 utilizes the dropout-based genes from scRNA-seq and ST data, along with spatial information of all spots as additional information. An autoencoder (AE) is used to obtain low-dimensional representations of the real and pseudo-spots, which reveals a nonlinear relationship among dropout genes. The classes are regarded as distinct cell types, and the output probabilities represent the composition of all cell types.

The input data for SD2 consists of the expression matrix and the adjacency matrix of the constructed graph. The input data is passed into a graph convolutional network (GCN) consisting of three convolutional layers. Furthermore, the network employs rectified linear unit (ReLU) as the activation function following each graph convolutional layer. Finally, the softmax activation function is used to normalize the output of three convolution layers.

By synthesizing the cells from different datasets, quantitative experiments under different resolutions of spots and measurements are conducted to evaluate the performance of SD2. This method employs uniform manifold approximation and projection to visually represent the two-dimensional projection of all spots with their highest-abundant cell type.

#### SpiceMix

SpiceMix introduces an interpretable technique that uses probabilistic, latent variable modelling to jointly analyze spatial information and gene expression from spatial transcriptome data. A probabilistic graphical model called NMF-HMRF is used by the SpiceMix algorithm to model spatial transcriptome data. This model provides a clear explanation for single-cell spatial transcriptome data, where each node in the graph represents an individual cell. With this paradigm, every node in the graph stands for a cell, making it easy to understand single-cell spatial transcriptome data. The NMF-HMRF model in SpiceMix uniquely combines the spatial modelling of the HMRF with the NMF formulation for gene expression, resulting in a single model for spatial transcriptome data.

SpiceMix simultaneously learns the intuitive metagenes of latent factors, the latent states for all nodes, and their spatial affinity when spatial transcriptome dataset is given as an input. This task is achieved by employing an alternating maximum a posteriori (MAP) optimization technique. Importantly, in SpiceMix, metagenes are an integral part of the model outcome, which presents a methodological advance in comparison to the calculation of spatially variable genes as a post-processing step in other methods.

The spatially variable metagene formulation is a key feature of SpiceMix, as it allows to describe the interaction between the intrinsic and spatial components of the transcriptome, rather than just the patterns of individual genes. SpiceMix incorporates the relationship between metagenes and continuous cell states as an essential aspect of its model formulation, setting it apart from other techniques.

SpiceMix’s methodology may also be well suited for multiomic data as it does with transcriptomic data. The scope of this algorithm can be enhanced that allows for progressive changes in the learned spatial patterns. Incorporating cell-to-cell interactions, the refined cell identities and learned spatial relationships of SpiceMix may be useful for studying other aspects of tissue dynamics. In sum, SpiceMix provides an effective platform for analyzing diverse types of multiomic and spatial transcriptome data.

The natural organization of spatial transcriptomics data enables graph-based methods (see Fig. 2B) to represent spots or cells as nodes connected by edges that encode spatial adjacency and/or transcriptional similarity. The DSTG achieves superior prediction results for complex tissues through its application of semi-supervised graph convolutional networks to pseudo-spots that link scRNA-seq and ST data. The SD2 method extends this principle through its direct modeling of dropout patterns and its implementation of graph embeddings which adds spatial proximity information to improve technical noise resistance while preserving detailed resolution. The design of SpiceMix differs from other methods, yet it uses NMF-HMRF to link gene expression factors with spatial relationships, which produces metagenes that represent both continuous states and spatial domains. The methods show how graph structures enable deconvolution through their ability to detect non-linear relationships and their use of spatial data to uncover tissue structures at multiple organizational scales. These methods provide two main advantages because they enable researchers to analyze spatial patterns and intricate non-linear relationships that exceed basic linear combination models. The method requires high computational resources and depends on the selection of graph construction parameters and pseudo-spot generation which can produce biased results. The graph-based approach enables direct spatial context integration into deconvolution, which produces better biological understanding of tissue architecture.

### Optimal transport-based

#### SpaOTsc

SpaOTsc stands for the spatial optimal transport for single-cell data and uses scRNA-seq data to create a method that reconstructs spatial features by analyzing a minimal set of spatial marker genes. The main concept of SpaOTsc is to build spatial metric for each cell by establishing an optimal mapping between scRNA-seq and spatial imaging data.

The first step of SpaOTsc creates spatial connections between cells from scRNA-seq and imaging data. The algorithm uses structured and unbalanced optimal transport methods to establish flexible data mapping between two imbalanced distributions. The method creates probabilistic spatial locations for individual cells by linking scRNA-seq data cells to their corresponding positions in spatial imaging data.

The optimal transport mapping enables SpaOTsc to calculate spatial distances between cells through their spatial probability distribution analysis. The generated distance matrix of cells forms a spatial metric space that embeds single cells into their spatial environment.

SpaOTsc establishes intercellular communication networks through probability distributions of sender and receiver cells, which are derived from gene expression profiles using this spatial metric. The sender distributions reflect ligand gene expression levels and the receiver distributions reflect corresponding receptor and downstream gene expressions.

SpaOTsc uses random forest models (decision trees ensemble) as a crucial step to determine the spatial extent of specific signaling events. Random forest models predict downstream gene expression outcomes through the analysis of ligand expression at different spatial ranges along with receptor expression weights. This process enables the identification of optimal signaling distances.

The analysis of SpaOTsc extends to study how genes in different cells interact through spatial distance. Through partial information decomposition the method evaluates the distinctive information one gene expression provides about another gene in adjacent cells. Through this method researchers can discover new gene regulatory pairs that exist between cells that are spatially distant.

SpaOTsc ensures the validity of its reconstructed spatial gene expression patterns through cross-validation and uses Spearman’s correlation coefficient and the area under the receiver operating characteristic curve (AUC) and root-mean-square error (RMSE) as evaluation metrics. The tool provides visualization and clustering methods for space-constrained analysis, which helps researchers compare their predicted spatial patterns to actual experimental data. SpaOTsc uses single-cell data to generate tissue spatial patterns and simultaneously reveals the spatial communication networks between cells in tissues.

#### novoSpaRc

The novoSpaRc computational system spatially reconstructs single-cell gene expression data through a probabilistic method that matches individual cells to their tissue locations. The structural correspondence hypothesis serves as the fundamental principle behind novoSpaRc since it states that cells positioned near each other tend to show matching gene expression profiles.

The primary objective of novoSpaRc involves generating a probabilistic spatial framework for cell mapping onto pre-defined target space in order to analyze tissue-specific gene expression patterns. The two essential inputs required for novoSpaRc to function are gene expression matrices and target spatial coordinates. The first necessary input consists of a gene expression matrix, which contains RNA counts or normalized gene expression data measured at the single-cell level. Second necessary input for novoSpaRc includes the target space, which contains physical tissue coordinates structured as 1D, 2D or 3D. Users have the option to include an atlas expression dataset, which contains known spatial expression patterns for marker genes to improve reconstruction quality.

The computational workflow of novoSpaRc begins after receiving the required inputs. The system generates three unique cost matrices, which consist of a cell–cell expression distance matrix based on k-nearest-neighbor (kNN) graphs in gene expression space, a location–location physical distance matrix from kNN graphs in coordinate space and an optional atlas-based cell–location distance matrix for marker-gene expression comparison.

The OT matrix computation follows in novoSpaRc for assigning cells to tissue locations probabilistically. The OT problem handled by novoSpaRc seeks to optimize a total function while satisfying conditions based on marginal distribution requirements. The overall objective function integrates two vital components, which include the Gromov–Wasserstein (GW) discrepancy together with Euclidean distances. The GW discrepancy functions to measure differences in pairwise distances that exist between cell–cell expression space and location–location physical space so that structural correspondence can be maintained. The mapping requirements are supported by this term to preserve gene expression structures that match tissue spatial arrangements. The discrepancy measurements based on Euclidean distances evaluate the differences between marker-gene expressions of cells and their reference atlas-derived spatial positions to produce mappings that follow established spatial expression patterns.

The OT solution in NovoSpaRc operates through two main parameters, which are $alpha (\alpha )$ and $epsilon (\varepsilon )$. The alpha value determines the weight between structural correspondence and atlas-based spatial information in novoSpaRc with $\alpha =0$ using GW discrepancy exclusively and $\alpha =1$ using atlas information exclusively. The OT solution uses intermediate alpha values to combine both approaches. The epsilon parameter regulates the entropy regularization process which generates more specific deterministic mappings when set to low values and produces more spread-out probabilistic mappings when set to higher values.

The final output of novoSpaRc consists of a probabilistic transport matrix, which shows the mapping likelihood between cells and locations, and a spatially predicted gene expression matrix that displays reconstructed tissue location gene expression patterns. NovoSpaRc produces fixed results from the algorithm because it works deterministically for identical inputs and parameter values. This algorithm applies geometric cell expression space structures against tissue physical locations to decrease dependence on reference atlases yet allows their use when available. The novoSpaRc framework allows users to estimate parameters such as effective neighborhood size and cell density to apply as biological prior knowledge that improves reconstruction quality.

The distribution-matching problem enables optimal transport-based methods to link gene expression similarity with physical spatial constraints through cell-to-location assignment. SpaOTsc uses both structured and unbalanced OT methods to match scRNA-seq data with restricted spatial information, which enables the generation of spatial metrics for cell–cell communication and gene–gene regulatory flow inference. The structural correspondence hypothesis allows NovoSpaRc to produce tissue maps by performing Gromov–Wasserstein expression and spatial distance alignment and using optional atlas-derived prior information when available. The two methods perform atlas-free reconstruction while they utilize prior knowledge to improve mapping performance when prior information is available. The system demonstrates three main advantages through its ability to handle multiple data types and its capability to map cell locations at high resolution and its probabilistic transport matrix analysis for interpretation. The implementation of these advantages requires handling three main difficulties, which include high computational requirements and the need to optimize hyperparameters and the risk of obtaining biased results when spatial references are insufficient. The combination of OT-based methods with regression and probabilistic and graph frameworks produces a mathematically (illustrated in Fig. 2C) sound system to merge scRNA-seq and spatial data analysis, which achieves peak performance when structural alignment exists but needs biological context adjustments.

### Deep learning and LLM-based

#### Tangram

Tangram exploits a deep learning framework to tackle two key challenges in spatial transcriptomics: (1) learning maps of spatial gene expression at the single-cell resolution, and (2) relating these maps with histological and anatomical information from the same specimens. It enables genome-wide spatial mapping from limited gene panels, corrects noisy or low-quality spatial measurements, and generates robust spatial maps of diverse cell types.

The algorithm requires single-cell or single-nucleus RNA sequencing (sc/snRNA-seq) data and spatial profiling data from the same region or tissue type, obtained by spatial methods like MERFISH, smFISH, STARmap, ISH, or Visium. The essential criterion is that the two modalities possess a minimum subset of shared genes. Tangram voxelizes the spatial data—where each voxel corresponds to a location such as a Visium spot or MERFISH cell—and aims to learn a probabilistic mapping from cells to voxels.

After randomly positioning the sc/snRNA-seq profiles in space, Tangram calculates an objective function that reflects the spatial correlation between each gene in both the sc/snRNA-seq data and the ST data. Optimizing the total spatial correlation across the common genes is achieved by spatially rearranging the sc/snRNA-seq profiles. The resulting mapping infers a high-resolution spatial transcriptome, with single-cell resolution and full transcriptome coverage, even if the original spatial data were coarse or limited to a subset of genes. Tangram produces a probabilistic mapping matrix, where each entry represents the likelihood of assigning a single-cell RNA-seq profile to a given spatial voxel. This probabilistic output captures uncertainty and allows for soft assignments of cell types. When spatial technology supports single-cell resolution (e.g., MERFISH or STARmap), a deterministic mapping can be derived by selecting the highest-probability assignment per voxel, effectively yielding a one-to-one match between cells and spatial locations.

The algorithm spatially aligns the sc/snRNA-seq data using nonconvex optimization. The spatial alignment is evaluated by employing two similarity functions: Kullback–Leibler (KL) divergence compares cell-density distributions and voxel-based similarity compares gene expression; both similarity functions are used to reward the spatial alignment. Specifically, the KL divergence penalizes mismatch in cell density between predicted and observed voxels, while cosine similarity is applied across both genes (global expression similarity) and voxels (local gene profile similarity). There are no hyperparameters in Tangram, and it can efficiently map a large number of cells in a minimum time span using only one GPU.

The analysis performed by Tangram on a measured subset of genes can be extended to genome-wide profiles to map the location of cells of interest and correct low-quality spatial measurements. Tangram learns a filter vector to select an optimal subset of cells when the number of sc/snRNA-seq cells exceeds the number of spatial voxels. Additionally, it incorporates a computer vision module that leverages histology data and maps it to anatomical positions in an existing common coordinate framework (CCF) in the brain. This enables the integration of cellular and anatomical scales, even in the absence of additional annotation.

The module consists of a Siamese neural network for depth prediction and a U-Net–based segmentation model for anatomical region identification. The system performs histology section alignment to anatomical atlases through automated processes that eliminate the need for manual landmarking. The system detects and corrects both low-confidence and noisy gene expression measurements to improve spatial pattern interpretability and robustness.

Tangram enables the creation of spatial patterns from multimodal single-cell profiles including RNA expression data and ATAC data. Tangram uses SHARE-seq data to create spatial RNA maps, which enable the prediction of ATAC patterns at single-cell resolution for spatial multi-omic profiling.

#### TransformerST

TransformerST is an unsupervised deep learning transformer-based architecture that performs super-resolution analysis as well as tissue type identification in spatial transcriptomics (ST) data. The method operates independently from single-cell RNA sequencing (scRNA-seq) references for deconvolution purposes. This system merges spatial gene expression data with histology imaging information through the synergistic approach. The algorithm performs its initial operation by selecting image patches around spatial spots before it applies a vision transformer (ViT) [36] to process both histological and genetic information. The integrated system maintains precise preservation of morphological context together with molecular signals, which enables the detection of subtle tissue structure variations.

Next, TransformerST generates a spatial graph with nodes representing spatial spots, which connect through edges that represent both physical adjacency and gene-image feature similarities obtained from the ViT. The graph transformer system applies masking along with multi-head attention mechanisms to its graph transformer model for analyzing spatial relationships between neighboring spots. The spatial learning process at this stage enables the discovery of unique cell-type characteristics while minimizing the effects of identical or overlapping cellular elements. The spatial embedding receives repeated refinements through unsupervised clustering by using KL divergence to select cluster assignments that maintain local structural integrity and global coherence.

The cross-scale internal graph network in TransformerST enables the advancement of gene expression information from spot-based to single-cell analysis. The method achieves super-resolved reconstruction through a process that transforms high-density image sections within each spot into their parent spot embeddings and then combines them via a similarity-weighted operation. Through this process dense expression maps can be produced for tissue sections without needing explicit single-cell labels. The final stage of TransformerST performs high-dimensional gene expression reconstruction through variance-aware training, which stabilizes expression values of genes that exhibit different variability levels. The system design enhances spatial resolution while offering scalable performance and platform compatibility for Visium and STOmics and precise detection of fine-grained tissue structures.

Deep learning and transformer-based frameworks introduce spatial deconvolution to a new level through their application of computer vision and natural language processing architectures. The Tangram system employs non-convex optimization techniques and neural networks to analyze sc/snRNA-seq data against spatial measurements, which produces detailed maps that combine histological characteristics. The system demonstrates three main advantages through its ability to process large cell numbers and its capability to handle multiple data types including RNA and ATAC and histology data and its output of probabilistic cell-to-voxel assignments. The TransformerST model operates independently of references because it unites ViT with graph transformers to extract knowledge from gene expression and histology images which results in high-resolution tissue maps and reveals hidden microanatomical details. Together, these approaches (see Fig. 2D) demonstrate the capacity of modern deep models to integrate diverse data modalities, enhance spatial resolution beyond experimental limits, and maintain robustness to noise and incomplete references. Transformers based architectures need large GPU resources and memory space for training and require substantial data to achieve reliable generalization but their interpretability remains limited compared to probabilistic and regression-based models. The deployment of models in practice depends on transfer learning and self-supervised pretraining and data augmentation techniques and engineering methods including mixed-precision training and model distillation to minimize computational resources and memory usage. The combination of deep learning with transformer/LLM-inspired methods provides flexible and powerful solutions for uniting spatial and single-cell data while showing great potential for multimodal integration and detailed tissue reconstruction but their implementation needs evaluation of computational needs and dataset size and model interpretability.

## Discussion

Computational methods for spatial transcriptomics (ST) deconvolution have become essential in spatial biology since they allow researchers to predict cell compositions from differentially resolved transcriptomic data. The main computational challenge persists because researchers need to correctly distribute gene expression data obtained from spatially imprecise capture areas to their cell type origins. Several computational frameworks including probabilistic models and non-negative matrix factorization (NMF) along with graph-based techniques and optimal transport and deep learning approaches have appeared to solve this challenge over the past few years. The wide variety of tools demonstrates the mixed nature of spatial analysis data and biological questions, which makes it crucial to evaluate them both through theoretical analysis and practical usage in different contexts.

The main distinction between deconvolution methods stems from their requirement of single-cell RNA sequencing (scRNA-seq) reference data. The majority of current approaches use reference data from annotated single-cell profiles to determine cell-type proportions in each spatial unit. The use of single-cell data enables such methods to achieve better accuracy when they have corresponding references available because they can leverage extensive transcriptomic information. The probabilistic approaches Cell2location, DestVI, and RCTD demonstrate this methodology by modeling spatial gene expression observations through cell-type-specific profiles and they also estimate uncertainties. Reference-based methods face limitations when utilizing high-quality tissue-matched references because these references remain unavailable or poorly annotated in cancer studies and developmental biology research and non-model organism investigations. Reference-free approaches STdeconvolve, Berglund, and SpiceMix serve as solutions for identifying expression programs from spatial transcriptomic data when references are unavailable. The models provide flexibility in analyzing uncharted biological systems but their ability to deliver interpretable results and detailed resolution might be compromised.

The degree to which methods utilize direct spatial information stands as an essential factor for evaluation. The graph-based models DSTG and SD2 use spatial neighborhood relationships in their framework to enhance deconvolution results, particularly when analyzing tissues with defined architectural structures or specific localized microenvironments. The deconvolution process in optimal transport-based methods SpaOTsc and novoSpaRc involves aligning spatial data distributions with reference profiles to extract both global and local information. The deep learning models Tangram and TransformerST demonstrate strong flexibility because they allow the integration of multiple data modalities. The approaches show great potential for histology image and multi-omic spatial measurement applications but require higher computational resources and generate less interpretable results than statistical models.

Benchmarking studies [37–39] have demonstrated that deconvolution method evaluation requires assessment of both precision and stability and operational simplicity. The accuracy of deconvolution results depends heavily on three main factors including dataset resolution, the number of cell types and the occurrence of dropout events. The methods CARD and DestVI demonstrate reliable performance in both simulated and real-world datasets, but SpatialDWLS demonstrates better performance in simulated data before failing to analyze real tissue complexity. The ability of methods to withstand variations in input data such as gene number, resolution, and spot count remains crucial for maintaining reproducibility in downstream analyses. The adoption of these methods depends heavily on how well they integrate into research practices as well as their installation simplicity and computational performance and documentation quality. Research tools RCTD, SpatialDecon, and Cell2location receive high praise for their user-friendly features, which simplify their application across various research domains.

Multiple significant challenges continue to affect the field despite recent progress. The process of platform effects between ST and scRNA-seq systems creates systematic discrepancies, which make integration between the two systems challenging. The integration of ST and scRNA-seq data becomes complicated because of platform effects that stem from different gene capture efficiencies and normalization approaches and library preparation methods. Several methods try to resolve these effects through RCTD normalization but cell-type-specific biases persist as an open problem. The high dropout rates and low detection sensitivity that characterize Stereo-seq and other subcellular ST platforms together with high-resolution ST platforms restrict deconvolution reliability, particularly for rare cell types and genes with low expression levels. The combination of imputation methods with dropout-aware models provides partial remedies but researchers need complete frameworks that handle both biological and technical variability.

The field advances towards uniting three-dimensional data with multimodal information. The analysis will incorporate histological images and epigenetic marks and proteomic information in addition to transcriptomic profiles to enhance spatial resolution and biological context. The integration of transcriptomic data will increasingly depend on deep learning models that use transformers and attention mechanisms as their fundamental basis. Three-dimensional spatial modeling will need innovative deconvolution frameworks which maintain tissue layer continuity because researchers now perform serial sectioning and volumetric imaging. The modeling of continuous cell states instead of discrete cell types represents a promising direction in research, which approaches such as DestVI use to study cell-state gradients along with intra-type heterogeneity that traditional hard clustering methods fail to detect. The path toward transparent and reproducible research will depend on creating standardized benchmarking systems and curated datasets together with unified evaluation criteria accepted by the scientific community.

### Comparative performance and practical guidelines

Multiple critical elements emerge from benchmarking research (see Table 2) that distinguish various approaches from each other [37–39].

#### Accuracy

The probabilistic models Cell2location, DestVI, and RCTD demonstrate high accuracy performance when they have access to high-quality scRNA-seq reference data. The regression/NMF methods (e.g., SpatialDWLS, SPOTlight) generate effective results when data conditions are appropriate, yet their performance compromised when dealing with noisy or heterogeneous data.

#### Robustness to noise

Graph- and OT-based methods (DSTG, SpaOTsc, and novoSpaRc) show resilience when spatial priors are informative but may be sensitive to graph construction or hyperparameters. Dropout remains a major challenge across all classes.

#### Reference requirements

Reference-based methods produce detailed results, but they become ineffective when reference information is absent or does not match the data. The flexibility of reference-free methods (STdeconvolve, Berglund, and SpiceMix) increases, but their output becomes more challenging to interpret.

#### Use of spatial information

Graph-based and OT-based approaches explicitly incorporate spatial structure, which benefits tissues with strong spatial organization. Probabilistic and regression approaches often treat spots independently.

#### Computational scalability

The most efficient methods for computational scalability are regression/NMF, but probabilistic and OT-based models require GPU acceleration for moderate scaling and deep learning or specially transformer-based models need the most resources to perform deconvolution or multi-modal integration.

In conclusion, spatial transcriptomics technology measurements at the pixel level find their connection to biological discovery needs through computational deconvolution techniques. The methodological framework demonstrates the field’s advanced stage and its ability to support multiple application domains. The combination of improved computational sophistication and enhanced data integration and user-friendly design approaches makes it possible for this field to achieve major breakthroughs. Basic and translational research will achieve its full potential through continued methodological innovation and standardization and interpretability improvements.

## Scientific notations

### General notation

-  ${y}_{ij}$ Expression of gene $i$ in spatial spot/sample $j$ 
-  ${r}_{i}$ Gene-wise correction or normalization factor
-  ${\beta }_{k}$ Proportion (mixing fraction) of cell type $k$ 
-  ${\mu }_{ik}$ Mean expression of gene $i$ in cell type $k$, estimated from single-cell data
-  ${\rm X}_{ik}$ Single-cell UMI count data of gene $i$ in cells of type $k$

### Distribution notation

-  $NB$ Negative binomial distribution (models over-dispersed scRNA-seq data)
-  $Multinomial({\widehat{\theta }}_{ik})$ Multinomial distribution with topic/cell-type proportions
-  $LogNormal({\mu }_{ik}, {{\sigma }^{2}}_{ik})$ Log-normal distribution with log-space mean and variance

### Parameter symbols

-  ${\lambda }_{ik}, {p}_{ik}$ Estimated NB parameters—dispersion and success probability
-  ${{\sigma }^{2}}_{ik}$ Variance for log-normal or Gaussian distributions
-  ${\theta }_{ik}$ Multinomial probability vectors for gene-topic models
-  ${W}_{ik}, {H}_{ik}$ Factorized matrices from NMF
-  $AE$ Autoencoder-derived latent expression (e.g., SD^2^)

### Spatial and graph terms

- ${A}_{j{j}{\prime}}$ Adjacency matrix defining spatial neighborhood
-  $\phi$ Spatial correlation coefficient (CARD-specific)
-  ${W}_{j{j}{\prime}}$ Spatial weight or proximity between spot $j$ and $j{\prime}$ 
-  $\sum_{x}^{-1}$ Spatial affinity matrix (SpiceMix, HMRF)
-  $f(\cdot )$ Graph convolution over neighbors (DSTG, SD^2^)
-  ${f}_{attn}(\cdot )$ Attention-based transformation (TransformerST)

### Optimization terms

-  $\lambda$ Regularization coefficient (e.g., in AdRoit or Tangram)
-  $Reg(\beta )$ Regularization penalty (e.g., L2 norm on proportions)
-  ${w}_{i}^{C}, {w}_{i}^{S}$ Weights for variability and specificity (AdRoit)
-  ${\mathcal{L}}_{expression}, {\mathcal{L}}_{spatial}$ Expression/spatial loss balance (novoSpaRc)

### Mapping and transport terms

-  ${T}_{jk}$ Transport matrix from spatial spot $j$ to cell type $k$ (SpaOTsc)
-  ${N}_{j}$ Total transcript count in spot $j$ (RCTD)
-  ${\alpha }_{i} , {\gamma }_{i}$ Gene/platform-specific biases (RCTD)

## Data Availability

No datasets were generated or analysed during the current study.
